# Habitat Heterogeneity of Nitrogen and Phosphorus Cycling Functional Genes in Rhizosphere Microorganisms of *Pinus tabuliformis* in Qinling Mountains, China

**DOI:** 10.3390/microorganisms13061275

**Published:** 2025-05-30

**Authors:** Hang Yang, Yue Pang, Ying Yang, Dexiang Wang, Yuchao Wang

**Affiliations:** 1 College of Forestry, Northwest A&F University, Yangling 712100, China; yhang07@126.com; 2 College of Forestry, Hebei Agricultural University, Baoding 071000, China; pangyue1109@163.com; 3 Mudan District Agriculture and Rural Bureau of Heze City, Heze 274000, China; 13032350410@163.com; 4 Xi’an Botanical Garden of Shaanxi Province (Institute of Botany of Shaanxi Province), Xi’an 710061, China

**Keywords:** functional genes, habitat heterogeneity, ecological adaptability, *P. tabuliformis*, Qinling Mountains

## Abstract

Microbial functional genes serve as the core genetic foundation driving microbial ecological functions; however, its microbial functional gene composition across varied habitats and its ecological adaptation interplay with plants remain understudied. In this study, we investigated the *P. tabuliformis* rhizosphere microbial functional genes which are related to N and P cycles across ridge and slope habitats between different elevational gradients, analyzed their composition and abundance, and analyzed their responses to environmental factors. Results showed that slope habitats had a significantly greater abundance of N and P cycling functional genes compared to those of ridge counterparts (*p* < 0.05). Specifically, slope environments showed an enhanced gene abundance associated with denitrification, nitrogen fixation, nitrification, assimilatory/dissimilatory nitrate reduction, and nitrogen transport processes, along with the superior expression of genes related to inorganic/organic phosphorus metabolism, phosphorus transport, and regulatory gene expression. These nutrient cycling gene levels were positively correlated with soil nutrient availability. Our findings revealed distinct ecological strategies: Ridge communities employ resource-conservative tactics, minimizing microbial investments to endure nutrient scarcity, whereas slope populations adopt competitive strategies through enriched high-efficiency metabolic genes and symbiotic microbial recruitment to withstand resource competition.

## 1. Introduction

Soil microorganisms constitute fundamental components of soil ecosystems, playing pivotal roles in sustaining soil quality and ecological functionality [1,2]. Their metabolic activities are profoundly shaped by spatiotemporal dynamics [3], particularly within the rhizosphere—a critically dynamic interface exhibiting distinct physicochemical gradients and biological interactions compared to bulk soil [4,5]. This microenvironment harbors microbial communities with enhanced biodiversity, structural complexity, and metabolic versatility, driving intensive plant–microbe crosstalk. Such functional specialization empowers rhizospheric microorganisms to perform critical functions in plant growth promotion and ecosystem stabilization [6,7].

Microbial functional genes constitute the genetic cornerstone of soil ecological processes [8,9], governing microbial functions that critically influence plant growth and ecosystem adaptation [5]. Compared to microbial community structure, microbial functional traits demonstrate heightened sensitivity to environmental fluctuations [10], with rhizosphere gene expression being co-regulated by root exudates, nutrient availability, and abiotic factors [11,12,13]. As primary limiting nutrients, nitrogen (N) and phosphorus (P) fundamentally constrain the soil ecosystem’s productivity [14,15]. Deficiencies in these nutrients suppress plant growth, alter organ-specific nutrient allocation, and reduce rhizosphere N/P cycling gene abundance [16]. Ecological stoichiometry and homeostasis theory further reveal compensatory plant–microbe interactions under nutrient stress, manifested through the selective enrichment of rhizosphere microbial consortia which are specialized in nutrient acquisition [17,18]. Such adaptations restructure functional communities, modulating nutrient cycling gene abundance to optimize soil resource availability [19]. Generally, due to N and P cycling pathways having inherent metabolic connections in soil systems, environmental heterogeneity may decouple the coordinated regulation of N and P cycling functional genes [20,21]. More and more evidence establishes strong correlations between N and Pcycling functional gene abundance and coupling relationships and environmental parameters such as plant community and soil physicochemical properties, and habitat-driven shifts in plant strategies and environmental variables dynamically regulate these genetic profiles through tripartite plant–microbe–environment interactions [22,23]. However, in heterogeneous habitat conditions, the abundance dynamics of functional genes regulating the distinct cycling processes of nitrogen and phosphorus elements, the coupling relationships among genes across different processes, and their respective environmental drivers remain poorly researched.

*P. tabuliformis*, a dominant native conifer in northern China, exhibits distinctive zonal distribution patterns in the Qinling Mountains. As reported by Zhu et al. [24], this species predominantly forms ridge-top communities, while occurring as discrete patches within slope oak forests, creating characteristic pine–oak mosaic ecosystems. This spatially structured distribution across elevational gradients—where steep environmental transitions occur over limited distances [25,26]—positions *P. tabuliformis* as an ideal model for investigating species distribution drivers and ecological adaptation mechanisms. In addition, *P. tabuliformis* is also considered to have higher environmental adaptability by having larger basic niche and higher phenotypic plasticity [27], and having more frequent or novel interactions with soil functional microorganisms [28].

In this study, we examined *P. tabuliformis* communities across ridge and slope habitats between high and low elevational gradients in the mid-Qinling Mountains. We profiled rhizosphere microbial N and P cycling functional genes along these gradients and assessed their structural organization and environmental drivers. Through comparative analyses of heterogeneous habitats, we elucidated linkages between microbial gene architecture and host adaptive strategies under environmental filtering. Specifically, we hypothesized the following: (1) The composition and abundance of microbial functional genes associated with nitrogen and phosphorus cycling exhibit heterogeneity across different habitats; and (2) the abundance of microbial functional genes involved in nitrogen and phosphorus cycling exhibits positive correlations with soil nutrient content, reflecting strategic adjustments in host plants’ resource utilization under varying resource conditions.

## 2. Materials and Methods

### 2.1. Site Description

This study was conducted in the Huoditang forest region, in the middle of the Qinling Mountains of Shaanxi Province, China (NSTEC: 108°21′ E, 33°18′ N–108°29′ E, 33°28′ N). The climate is transitional between northern subtropical and warm temperate, with mean annual temperature and precipitation ranging from 8 to 10 °C, and 1000 to 1200 mm, respectively. In addition, 70% of the precipitation occurs between June and September. The dominant soil type is brown forest soil, with an average depth of 50 cm and a pH of 6.5. The vegetation in the study area is dominated by mixed-temperate coniferous and broadleaf forests and frigid coniferous forests, and the percentage of forest cover is approximately 93.8%. The dominant tree species are *Quercus aliena* var. *acuteserrata*, *P. tabuliformis*, *P. armandii*, *Tsuga chinensis*, and *Picea asperata*. Currently, 95% of the forest is secondary forest that was restored after heavy felling during the 1960s [29,30].

### 2.2. Experimental Design and Sampling

Chinese pine is one of the most widely distributed native conifer species in northern China and plays essential roles in soil and water conservation and biodiversity maintenance in temperate forest communities [31]. The distribution area of *Pinus tabuliformis* in the Qinling Mountains decreases gradually from east to west, and Huoditang, Ningshan County, on the southern slope of the mid-Qinling Mountains, is an important area where it is concentrated [32]. Zhu [24] and Xu [33] found that most *P. tabuliformis* individuals were zonally distributed on ridges or had small-area patches inlaid on the oak forest of the slope habitats, forming pine–oak mixed forests.

Within the distribution range of the *P. tabuliformis* population in the study area, two elevation (high and low elevations) gradients and two different habitats (slope and ridge habitats) were selected and 20 m × 20 m quadrats were established. To guarantee the difference in the above- and belowground communities of plots between altitude, the altitude difference in neighbor elevation gradients was not less than 400 m. Four replicates were prepared for each habitat of two elevation gradients.

The rhizosphere soil was collected from the fine roots of each plot using a sterile soft brush. After litter removal (stones and plant and animal residues), the rhizosphere soil was frozen and stored at −80 °C for soil microbial DNA extraction, and a total of 16 rhizosphere soil samples were collected. For bulk soil sampling, five replicate points were selected at the four corners and the center of the plot. After removing the litter layer, five soil samples (0–20 cm depth) were collected from each point using a 5 cm diameter stainless-steel auger and then fully homogenized to provide one composite sample per plot. A total of 16 bulk soil samples were collected. All soil samples were air-dried and stored at room temperature for physicochemical analysis after being sieved through a <2 mm mesh.

### 2.3. Soil Properties

Soil pH was measured using a pH meter after shaking a soil–distilled water (1:2.5, *w*/*v*) suspension for 30 min at 200 rpm. The soil organic carbon (TC) was measured using the K_2_Cr_2_O_7_ oxidation method. Soil total nitrogen (TN) was determined using a semi-automatic Kjeldahl apparatus after digestion with K_2_SO_4_:CuSO_4_·5H_2_O (10:1 *w*/*w*)-H_2_SO_4_, while soil total phosphate (TP) was determined by colorimetry with a UV spectrophotometer after digestion with HClO_4_-H_2_SO_4_. Soil nitrate nitrogen (NO_3_^−^-N) and ammonium nitrogen (NH_4_^+^-N) were determined using a continuous flow analyzer (Auto Analyzer 3-AA3, Germany) after extraction with KCl [3].

### 2.4. DNA Extraction and Bioinformatics Analysis

DNA extraction of *P. tabuliformis* rhizosphere soil samples was achieved according to the manufacturer’s kit (FastDNA™ Spin Kit for Soil). Following DNA extraction, DNA concentration and purity were quantified, and electrophoresis (voltage 5 V/cm for 20 min) was performed on 1% agarose gel carriers using NanoDrop2000 and TBS-380 instruments to obtain the mass and concentration of the soil DNA. Before constructing the Paired-End (PE) library, DNA was broken into fragments of approximately 400 bp using an ultrasonic fragmentation instrument Covaris M220. PE libraries were then generated according to the NEXTFLEX Rapid DNA-Seq Kit instructions. After the enrichment of the library template using a polymerase chain reaction (PCR) system to perform amplification, the final library was obtained by recovering the PCR product with magnetic beads [34]. In addition, PCR was processed according to the manufacturer’s kit (NovaSeq Reagent Kits/HiSeq X Reagent Kits). The adapter sequences at the 3′ and 5′ ends of the reads were quality clipped using the software fastp. Then, the sequences were processed using MEGAHIT (https://github.com/voutcn/megahit (accessed on 10 December 2022), version 1.1.2) and MetaGene to obtain optimized sequences for splicing assembly and gene prediction, respectively. The construction of non-redundant gene sets was achieved by clustering (parameters: 90% identity, 90% coverage) the predicted gene sequences of all samples using CD-HIT (http://www.bioinformatics.org/cd-hit/ (accessed on 10 December 2022), version 4.6.1). Using SOAPaligner software, the high-quality reads of each sample were compared with the non-redundant gene set separately (95% identity) to count the gene abundance information in the corresponding samples. Diamond, version 0.8.35) was used to align the amino acid sequence of the non-redundant gene set with the Kyoto Encyclopedia of Genes and Genomes (KEGG) database (BLASTP alignment parameter setting expectation e-value is 1 × 10^−5^) and obtain the function annotation through the taxonomic information database corresponding to the KEGG database. Gene abundance was normalized using RPKM (Reads Per Kilobase per Million mapped reads) to account for variations in gene length and sequencing depth. For each gene, RPKM values were calculated as follows: $RPKM=\frac{Number\;of\;reads\;mapped\;to\;the\;gene}{\left(Gene\;length\;in\;kilobases)*(Total\;mapped\;reads\;in\;millions\right)}$, where gene length was derived from reference databases. Then, the functional category abundances were determined through the aggregation of gene abundances linked to KEGG Orthology (KO) identifiers. The Majorbio Cloud Platform was used to provide the measured data to analyze various types of information [35,36,37].

### 2.5. Statistical Analyses

The raw differential expression analysis was performed by Majorbio Cloud Platform, which inherently applies Benjamini–Hochberg FDR correction (*q*-value < 0.05) to all gene-level comparisons as part of its standardized bioinformatics workflow. This step ensured that only genes passing the FDR threshold were reported as statistically significant. In downstream analyses, we focused on pre-filtered N and P cycling genes that achieved FDR-adjusted significance (*q* < 0.05) from the sequencing platform’s primary analysis. One-way analysis of variance (ANOVA) was used to evaluate differences in the abundance of soil microbes’ functional genes between different habitats; significant differences were determined at a 0.95 confidence level (*p* < 0.05). Pearson’s correlation analysis was used to analyze the relationship between gene abundance and environmental factors. All data were sorted by Microsoft Excel 2019. ANOVA and Pearson correlation analysis were conducted using SPSS 23.0, and all photos were plotted using Origin 2021 pro.

## 3. Results

### 3.1. Nitrogen and Phosphorus Cycling Functional Gene Composition in Different Habitats

N and P cycling process are understood at biochemical and regulatory levels. Key N cycle processes include the following: Nitrogen fixation: Conversion of inert N_2_ to bioavailable ammonia (NH_3_/NH_4_^+^). Organic N metabolism: Degradation of organic nitrogen compounds to NH_4_^+^. Nitrification: Stepwise oxidation of NH_4_^+^ to NO_2_^−^; and NO_3_. ANRA (assimilatory nitrate reduction to ammonium): NO_3_^−^; to NH_4_^+^ for biosynthetic use. DNRA (dissimilatory nitrate reduction to ammonium): Anaerobic NO_3_^−^; to NH_4_^+^ retention. Denitrification: Reduction of NO_3_^−^; to N_2_ gas. And key P cycle processes include the following: Regulation, inorganic P transport, organic P mineralization, and inorganic P solublization [38,39].

**Figure 1:**
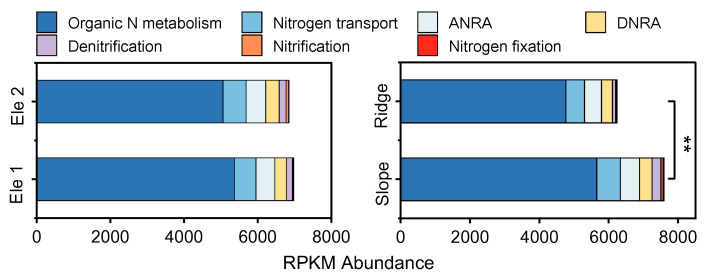
Composition characteristics of functional genes related to nitrogen cycling in rhizosphere soil of *P. tabuliformis* population in different habitats. “ANRA” represents assimilatory nitrate reduction to ammonium, “DNRA” represents dissimilatory nitrate reduction to ammonium, “**” represents significance (*p* < 0.01).

A comparative analysis was conducted on the abundance, composition, and variations in N and phosphorus P metabolic functional genes in the rhizosphere of *P. tabuliformis* populations across different habitats. The results revealed that the abundance of nitrogen metabolic functional genes in slope habitats was significantly higher than that in ridge habitats (*p* > 0.05), and abundance in low-elevation habitats (Ele1) was higher than that in high-elevation habitats (Ele2), but did not reach the significance level. Across both topographic and elevational gradients, genes associated with organic nitrogen metabolism exhibited higher abundance, whereas those linked to the nitrogen fixation, nitrification, and denitrification processes showed lower abundance (Figure 1).

**Figure 2:**
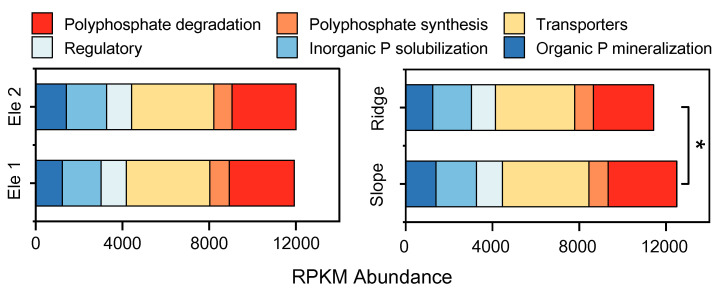
Composition characteristics of functional genes related to phosphorus cycling in rhizosphere soil of *P. tabuliformis* population in different habitats. “*” represents significance (*p* < 0.05).

The abundance of P metabolic functional genes showed no significant variation across elevational gradients; however, slope habitats exhibited significantly higher abundances compared to ridge habitats. Overall, genes associated with polyphosphate synthesis, phosphorus transport, inorganic phosphate solubilization, and organic phosphorus mineralization displayed higher abundances across both topographic and elevational gradients within the phosphorus metabolic functional gene pool (Figure 2).

### 3.2. Differences in the Abundance of Nitrogen Cycling Functional Genes Across Habitats

**Figure 3:**
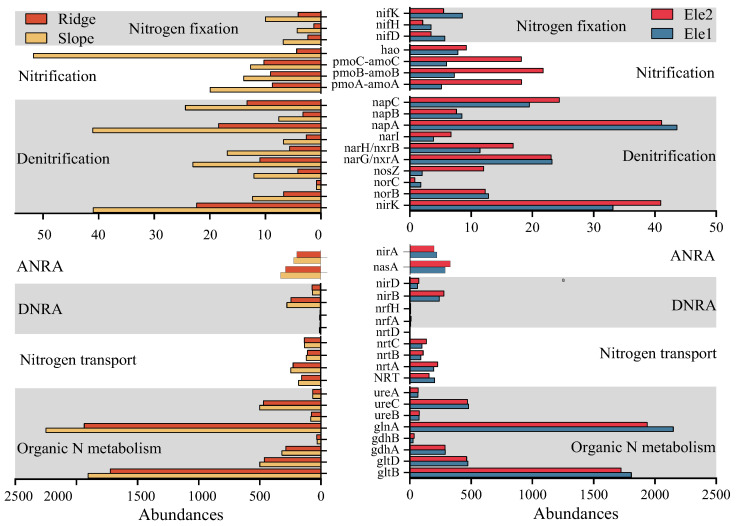
Histogram of nitrogen cycle functional genes.

**Figure 4:**
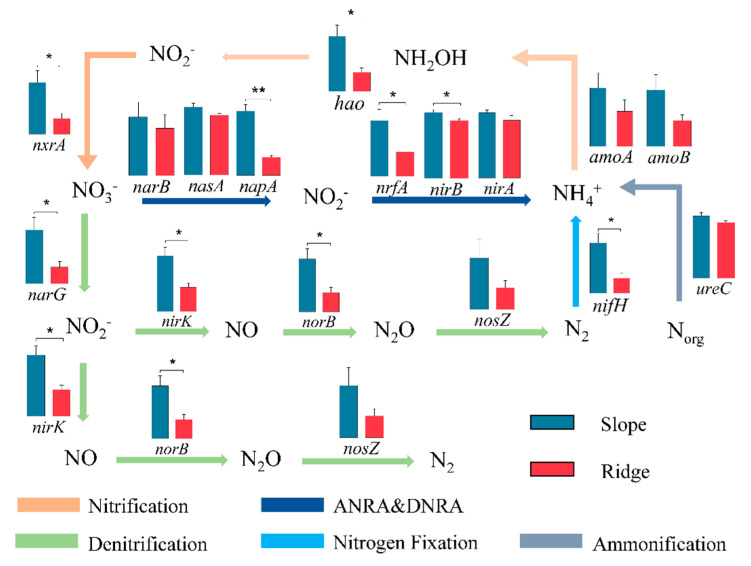
The differences in key genes in the process of the nitrogen cycle between topographic gradients. “*” represents significance (*p* < 0.05), “**” represents significance (*p* < 0.01).

**Figure 5:**
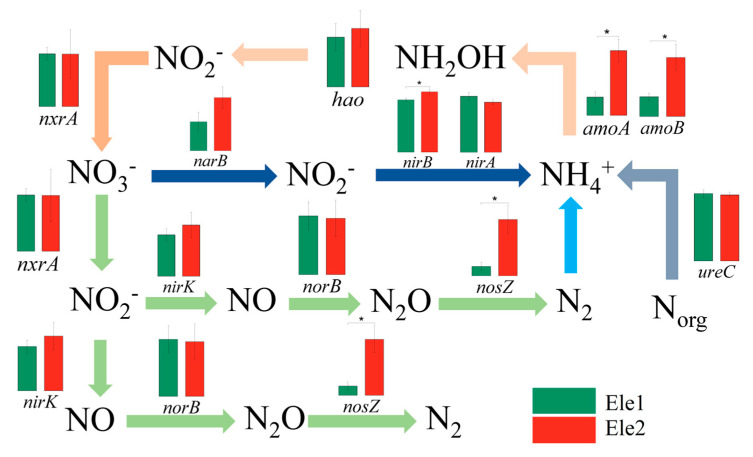
The differences in key genes in the process of the nitrogen cycle between altitude gradients. “*” represents significance (*p* < 0.05).

Functional gene differences in nitrogen metabolism processes among habitats are shown in Figure 3, Figure 4 and Figure 5. Along topographic gradients, slope habitats exhibited higher functional gene abundances for denitrification, nitrogen fixation, nitrification, assimilatory/dissimilatory nitrate reduction processes, and nitrogen transport compared to ridge habitats. Along altitudinal gradients, high-altitude habitats showed significantly lower abundances of functional genes related to nitrogen fixation and organic nitrogen metabolism than low-altitude habitats, while demonstrating elevated abundances for nitrification and assimilatory/dissimilatory nitrate reduction processes.

The analysis of key nitrogen metabolism genes revealed that along topographic gradients, slope habitats exhibited significantly greater abundances of functional genes including *nifH* (nitrogen fixation), *hao* and *nxrA* (nitrification), *narG*, *nirK*, and *norB* (denitrification), as well as *napA, nrfA*, and *nirB* (assimilatory/dissimilatory nitrate reduction) compared to ridge habitats. Along altitudinal gradients, high-altitude habitats showed markedly higher abundances of nitrification genes (*amoA, amoB, nxrA*), the denitrification gene *nosZ*, and assimilatory/dissimilatory nitrate reduction genes (*nasA, nirB*) than low-altitude habitats.

### 3.3. Differences in the Abundance of Phosphorous Cycling Functional Genes Across Habitats

The microbial phosphorus metabolism process mainly includes inorganic phosphorus metabolism, organic phosphorus metabolism, phosphorus transport, and regulation, and the abundance of microbial functional genes in each metabolic process is higher in the slope habitat than in the ridge habitat.

The abundance of inorganic phosphorus transporter genes such as *pit*, *ugpA*, *ugpB*, *ugpC*, and *ugpE* in the slope habitat was significantly higher than that in the ridge habitat. The abundance of the alkaline phosphatase gene *phoD* in the slope habitat was significantly higher than that in the ridge habitat during the mineralization of organic phosphorus. The abundance of the phosphorus regulatory gene *phoU* in the slope habitat was significantly higher than that in ridge habitat. Along the altitude gradient, the organic phosphorus mineralization and inorganic phosphorus metabolism gene abundance in high-altitude habitats was higher than that in low-altitude habitats. The abundance of *glpQ*, *ugpB*, *phnK*, *phnE*, *phnC*, *and phnD* genes in high-altitude habitats was higher than that in low altitude habitats. The abundance of the *PK*, *ppgK*, surE, and *ppk2* polyphosphate synthesis genes was higher in high-altitude habitats than in low-altitude habitats. The abundance of inorganic phosphorus transporter *pstA* and *pstS* genes in low-altitude habitats was significantly higher than that in high-altitude habitats. Organic phosphorus mineralization, including *phnG*, *phnH*, *phnI*, and other genes related to C–P lyase, was significantly higher in high-altitude habitats than in low-altitude habitats (Table 1).

### 3.4. Relationship Between the Functional Gene Abundance and Environmental Factors

Pearson correlation analysis was performed on the abundance of each microbial functional gene and environmental factors to explore the influencing factors on the abundance of functional genes related to nitrogen and phosphorus metabolism in the rhizosphere soil of the *P. tabuliformis* population. The nitrogen fixation gene abundance was significantly positively correlated with soil phosphorus content and soil pH, and negatively correlated with altitude and topography. The nitrification gene abundance was positively correlated with soil TC and TN content, and negatively correlated with NO^3−^-N content, and the abundance of the slope habitat was greater than that of the ridge habitat. The denitrification gene abundance was significantly positively correlated with soil nitrogen and phosphorus content, and its abundance decreased significantly from slope to ridge habitat. Assimilation and dissimilatory nitrogen reduction process gene abundances were significantly positively correlated with soil nitrogen. The nitrogen transport gene abundance was positively correlated with soil nitrogen and phosphorus content, while the abundance of genes in the slope habitat was significantly higher than that in the ridge habitat. The abundance of soil organic nitrogen metabolism functional genes was significantly positively correlated with soil phosphorus content and NO^3−^-N, and the abundance in the slope habitat was significantly greater than the ridge habitat.

**Figure 6:**
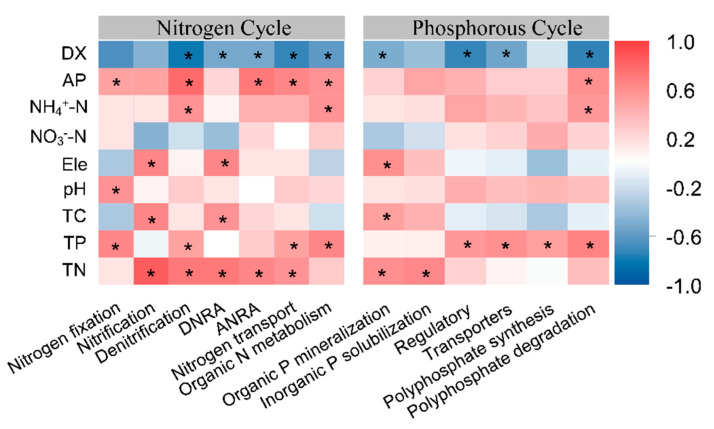
The correlation between the function of the nitrogen and phosphorus cycle genes and environmental factors. “*” represents significance (*p* < 0.05).

The correlation between the abundance of phosphorus metabolism genes and environmental factors is shown in Figure 6: The abundance of organic phosphorus mineralization and inorganic phosphorus dissolved genes was significantly positively correlated with soil nitrogen content. The abundance of phosphorus metabolism regulatory genes, transport genes, and polyphosphate synthesis genes were significantly positive correlated with soil phosphorus content, and the abundance of each functional gene was significantly higher in the hillside habitat than that in the ridge habitat.

## 4. Discussion

### 4.1. Nitrogen and Phosphorus Cycling Functional Gene Composition in Different Habitats

The rhizosphere, a critical interface between plants and their surrounding environment, is shaped by dynamic interactions between plant activity and environmental factors [5]. Rhizosphere microbial communities exhibit a dual ecological role: while dependent on root exudates for sustenance, they actively participate in nutrient cycling (particularly nitrogen and phosphorus), enhance soil nutrient availability, and ultimately promote plant growth [40]. As essential macronutrients, nitrogen (N) and phosphorus (P) frequently act as limiting factors for plant growth [38,41], with soil functional genes serving as key drivers of their bioavailability [42]. In nitrogen metabolism, functional genes associated with nitrogen fixation, nitrification, denitrification, and ammonification regulate nitrogen supply, leaching, and transformation [43].

In this study, a comparative analysis was conducted on the abundance, composition, and variations in N and phosphorus P metabolic functional genes in the rhizosphere of *P. tabuliformis* populations across different habitats. We found that the N metabolic functional gene abundance in slope habitats was significantly higher than that in ridge habitats (*p* > 0.05), and the abundance in Ele1 was higher than that in Ele2 but did not reach a significant level. Notably, slope habitats exhibited higher abundances of organic nitrogen assimilation genes and nitrogen transport genes compared to ridge habitats, whereas genes linked to nitrogen fixation, nitrification, and denitrification showed lower abundances. These findings suggest that *P. tabuliformis* populations predominantly rely on bioavailable organic nitrogen derived from decomposing biological residues as their primary nitrogen source [44]. The dominance of the organic nitrogen metabolism genes in slopes aligns with *P. tabuliformis*’s reliance on organic nitrogen from decomposing plant residues, a strategy critical for sustaining growth in nutrient-competitive environments [45]. Conversely, ridge habitats showed elevated *nirK* gene abundance, consistent with their lower soil moisture and higher nitrate leaching risks, reflecting adaptive nitrogen conservation under resource scarcity [46].

The P metabolic functional gene abundance in slope habitats was significantly higher than that in ridge habitats (*p* > 0.05). In P cycling, functional genes governing organic phosphorus mineralization, inorganic phosphorus dissolution, and polyphosphate degradation demonstrated habitat-specific patterns. Slope microbial communities were enriched in *phoD* and *ppx* genes, facilitating efficient phosphorus mobilization [47]. In contrast, ridge populations exhibited lower phosphate transport gene expression, likely reallocating metabolic resources to stress-tolerance mechanisms. Spatially, while Ele1 sites showed numerically higher gene abundances than Ele2, these differences lacked statistical significance, which emphasizes that topographic heterogeneity (slope vs. ridge), rather than elevation alone, drives functional gene divergence. This aligns with soil physicochemical profiles: slope soils had a higher nutrient- and water-holding capacity, creating a microenvironment conducive to microbial activity and nutrient retention [48]. Collectively, these findings reveal a mechanistic link between functional gene allocation, microbial recruitment, and edaphic adaptation. Slope populations leverage high-efficiency nutrient metabolism genes (e.g., *phoD*) and symbiotic microbial networks to outcompete neighbors, whereas ridge populations prioritize survival through metabolic austerity and stress-responsive traits. This resource-acquisition vs. stress-tolerance trade-off, usually mirrored in root exudate chemistry, reflects how plants orchestrate gene–microbe–soil interactions to optimize fitness across ecological gradients. Future studies on plant adaptation should prioritize the interactions between root metabolites and microbial communities.

### 4.2. Relationship Between the Functional Gene Abundance and Environmental Factors

To elucidate the relationship between the abundance and structure of N and P cycling functional genes in rhizosphere microorganisms and the ecological adaptation strategies of *P. tabuliformis* populations across heterogeneous habitats, a comparative analysis was conducted to assess differences in functional gene abundance among habitats and their correlations with environmental factors. Results showed that the nitrogen fixation gene abundance was significantly positively correlated with soil phosphorus content and soil pH, and negatively correlated with altitude and topography. The nitrification gene abundance was positively correlated with soil TC and TN content, and negatively correlated with NO_3_^−^-N content. The abundance of organic phosphorus mineralization and inorganic phosphorus dissolved genes was significantly positively correlated with soil nitrogen content. The abundances of phosphorus metabolism regulatory genes, transport genes, and polyphosphate synthesis genes were significantly positively correlated with soil phosphorus content. Studies have shown that plants exhibit divergent ecological strategies across environmental gradients: conservative resource retention dominates under scarcity, while competitive acquisition prevails in nutrient-rich habitats [49,50]. Our results suggest that *P. tabuliformis* populations adopt a competitive strategy at the functional gene level to enhance rhizospheric nutrient acquisition in slope habitats, whereas in nutrient-poor ridge and high-elevation habitats, they shift to a defensive and conservative strategy by reducing the investment in recruiting functional microorganisms specialized in rhizosphere nutrient uptake [51]. Notably, nitrogen acquisition genes correlated positively with soil available P, suggesting synergistic N-P mobilization in nutrient-rich slopes [52]. Conversely, ridge populations reduced the investment in nitrogen fixation and phosphate transport, likely reallocating resources to stress-tolerance traits under nutrient scarcity [53]. Collectively, these multilevel adaptations—from gene regulation to microbial recruitment—highlight how edaphic constraints shape evolutionary trade-offs between nutrient acquisition and stress resilience in forest ecosystems.

This study characterized the spatial heterogeneity of nitrogen and phosphorus cycling genes in *P. tabuliformis* rhizosphere microbiomes using metagenomic data. However, functional gene abundance demonstrated no positive correlation with transcriptional activity [54], suggesting limitations in extrapolating plant–microbe ecological adaptation solely from abundance metrics. To address this constraint, future studies should implement multi-omics approaches—such as targeted gene knockout and stable isotope probing (SIP)—to validate functional dynamics [55]. Additionally, spatial sampling variability and uncontrolled environmental covariates may confound gene expression profiles. Consequently, integrating field observations with controlled laboratory experiments is critical to establishing causal relationships among gene abundance, metabolic activity, and ecological functionality.

## 5. Conclusions

The comparative analysis of rhizosphere functional genes involved in nitrogen (N) and phosphorus (P) metabolism across Pinus tabuliformis habitats revealed a significantly higher abundance of nutrient acquisition genes in slope habitats compared to ridge habitats (*p* < 0.05), with gene abundances positively correlated with soil nutrients. These findings demonstrate a habitat-specific adaptive dichotomy: Ridge populations adopt a resource-conservative strategy, minimizing investment in microbial-mediated nutrient acquisition to prioritize survival under nutrient scarcity. In contrast, slope populations employ a competition-oriented strategy, enriching high-efficiency nutrient metabolism genes and recruiting symbiotic microbes to counter resource competition pressures. This gene–microbe–host triad alignment reflects ecological trade-offs in plants and microorganisms, reflecting the adaptive processes of plants in heterogeneous environments.

## Figures and Tables

**Table 1:** Comparison of the abundance differences of phosphorus cycle functional genes between different habitats.

Genes		Geography		Elevation	
		Slope	Ridge	Ele1	Ele2
Regulatory genes	*phoB*	204.72 a	179.04 a	188.88 a	194.88 a
	*phoR*	535.62 a	488.44 a	513.51 a	510.56 a
	*phoP*	13.95 a	13.02 a	13.96 a	13.02 a
	*phoU*	449.10 a	421.67 b	440.13 a	430.64 a
Primary inorganic P transporters	*pstA*	361.60 a	326.45 a	371.50 a	316.56 b
	*pstB*	490.82 a	428.30 a	475.59 a	443.44 a
	*pstC*	421.14 a	401.60 a	430.13 a	392.61 a
	*pstS*	726.31 a	747.81 a	763.23 a	710.89 b
	*pit*	500.17 a	429.74 b	495.69 a	434.22 a
Secondary inorganic P transporters	*phnC*	107.69 a	109.00 a	88.52 b	128.18 a
	*phnD*	141.19 a	135.93 a	125.41 a	151.72 a
	*phnE*	182.62 a	166.87 a	153.08 b	196.41 a
Glycerol-3-hosphate transporter	*ugpA*	103.11 a	71.67 ab	83.61 a	91.17 a
	*ugpB*	256.85 a	187.96 b	214.76 a	230.05 a
	*ugpC*	136.72 a	98.84 b	120.88 a	114.67 a
	*ugpE*	104.73 a	82.44 b	90.46 a	96.71 a
Alkaline phosphatase	*phoA*	80.10 a	58.58 a	67.76 a	70.93 a
	*phoD*	398.51 a	320.52 b	336.93 a	382.10 a
Inorganic P solublization	*gcd*	758.14 a	765.52 a	728.62 a	795.04 a
	*ppa*	223.32 a	225.79 a	245.29 a	203.82 b
	*ppx*	672.01 a	592.67 b	633.53 a	631.14 a
C–P lyase	*phnG*	45.86 a	44.91 a	38.07 b	52.70 a
	*phnH*	47.39 a	50.83 a	40.09 b	58.13 a
	*phnI*	95.84 a	86.24 a	83.68 b	98.41 a
	*phnJ*	70.44 a	67.69 a	68.11 a	70.02 a
	*phnL*	57.40 a	57.52 a	53.83 a	61.09 a
	*phnM*	155.61 a	144.59 a	143.60 a	156.60 a
	*phnN*	46.55 a	42.21 a	41.31 a	47.45 a
	*phnP*	167.34 a	152.23 a	148.53 a	171.04 a
	*phnK*	60.29 a	62.82 a	55.13 a	67.97 a
	*phnF*	47.28 a	43.89 a	50.76 a	40.40 a
Organic P mineralization	*Phy*	0.48 b	4.04 a	1.73 a	2.79 a
	*phnX*	14.69 a	17.08 a	16.93 a	14.84 a
	*phnW*	80.49 a	68.22 a	68.67 a	80.04 a
	*phnA*	94.85 a	96.37 a	86.24 b	104.98 a
	*phoN*	21.45 a	20.48 a	18.79 a	23.14 a

Note: A different letter within a column represents the significant differences (*p* < 0.05) of the functional genes between different habitats.

## Data Availability

The data presented in this study are available on request from the corresponding author (email: wangdx66@126.com) or the first author (email: yhang07@126.com) of this article.
